# Health‐Related Quality of Life of Adults With Long COVID: A Cross‐Sectional Study in Primary Care

**DOI:** 10.1111/jocn.70383

**Published:** 2026-05-30

**Authors:** Assel Muratovna Shigayeva Ferreira, Flávia Emília Leite de Lima Ferreira, João Agnaldo do Nascimento, André Luís Bonifácio de Carvalho, Caio César Ferreira Alverga, Leandro Pernambuco

**Affiliations:** ^1^ Center of Exact and Natural Sciences Federal University of Paraiba Joao Pessoa Brazil; ^2^ Center of Health Sciences Federal University of Paraiba Joao Pessoa Brazil; ^3^ Center of Medical Sciences Federal University of Paraiba Joao Pessoa Brazil; ^4^ Center of Health Sciences Federal University of Pernambuco Recife Brazil

**Keywords:** COVID‐19, EQ‐5D‐5L, health‐ related quality of life, long COVID, primary care

## Abstract

**Aims:**

To evaluate the health‐related quality of life (HRQOL) of adults with Long COVID 2 years and beyond after COVID‐19 illness.

**Design:**

Cross‐sectional study.

**Methods:**

Health status was assessed using the EQ‐5D‐5L instrument among 226 adults diagnosed in primary care with mild‐to‐moderate COVID‐19 during the 2021 pandemic. Data were collected through a cross‐sectional survey using a standardized questionnaire with a set of validated clinical outcomes for Long COVID. The sample consisted of adults aged ≥ 18 years who attended the specified ambulatory settings, tested positive for SARS‐CoV‐2, and agreed to be interviewed; the response rate was 70%. Health utility scores were compared between adults with and without Long COVID. Multivariate logistic regressions were applied to investigate the relationship between Long COVID and health‐related quality of life outcomes.

**Data Sources:**

Primary data were collected from six public Family Health Care Units in João Pessoa, Brazil, between May 2023 and July 2024.

**Results:**

Adults with Long COVID had statistically significantly lower median utility scores (0.784, IQR: 0.633–0.902) than those without persistent symptoms (1.0, IQR: 0.877–1.0). Poorer HRQOL was more evident among women, older adults, non‐White individuals, participants with pre‐existing chronic diseases, and those with lower educational attainment. Long COVID was associated with impairments in anxiety/depression, pain/discomfort and usual activities.

**Conclusion:**

Adults with Long COVID experienced poorer HRQOL 2 years or longer after mild‐to‐moderate infection compared with those without persistent symptoms, regardless of sex, age, ethnicity, education level or comorbidities. These findings support the implementation of targeted interventions and rehabilitation services in primary care for individuals experiencing long‐term health problems following COVID‐19 illness.

**Implications for the Profession and/or Patient Care:**

Identifying adults at greater risk of persistent health impairments following COVID‐19 may help health professionals, caregivers and policymakers better address the aspects of patients' lives that lack quality and develop a multidisciplinary approach in primary care to managing this condition.

**Impact:**

- What problem did the study address?
  ○ This study examined the association between persistent symptoms 2 years or longer after non‐severe COVID‐19 illness and health‐related quality of life.
- What were the main findings?
  ○ Long COVID was associated with poorer health‐related quality of life, particularly in the domains of anxiety/depression, pain/discomfort and usual activities.
- Where and on whom will the research have an impact?
  ○ The findings highlight the need for multidisciplinary management of long‐term health problems among adult COVID‐19 survivors in primary care.

**Reporting Methods:**

The STROBE checklist was followed.

**Patient or Public Contribution:**

No patient or public contribution.

## Key Points

- Adults with Long COVID experienced significantly poorer health‐related quality of life 2 years and beyond after non‐severe COVID‐19 illness.
- Persistent post‐COVID 19 symptoms were strongly associated with anxiety or depression, pain or discomfort, and limitations in usual activities.
- Women, older adults, non‐White individuals, people with chronic conditions and those with lower educational attainment chowed greater reduction in health‐related quality of life.
- The findings support the need for multidisciplinary interventions and rehabilitation services for Long COVID management in primary care settings.

## 1 Introduction

While many individuals recover to their baseline health following coronavirus disease 2019 (COVID‐19), a substantial number of adults experience symptoms that persist well beyond the acute phase of infection (Rajan et al. 2021). This phenomenon, commonly referred to as Long COVID, can affect anyone exposed to the virus, regardless of age or the severity of their initial symptoms (Soriano et al. 2022).

The World Health Organization (WHO) defines quality of life as ‘individuals' perceptions of their position in life in the context of the culture and value systems in which they live and in relation to their goals, expectations, standards and concerns’ (World Health Organization Quality of Life Assessment [WHOQOL] 1995). The COVID‐19 pandemic has had a significant negative impact on population well‐being (Rajan et al. 2021; Poudel et al. 2021). However, the long‐term effects of COVID‐19 on health‐related quality of life (HRQOL) among survivors have been less documented (Sanchez‐Ramirez et al. 2021; Ma et al. 2022; Dorri et al. 2021; Kim et al. 2023).

A meta‐analysis examining persistent effects of COVID‐19 reported a substantial reduction in HRQOL among 52% of individuals beyond 3 months after infection (Sanchez‐Ramirez et al. 2021). Another meta‐analysis involving more than 10,000 adults with Long COVID found a significant decline in HRQOL 1 year after the acute infection (Ma et al. 2022). In addition, pooled estimates from 21 cohort studies involving 49,650 COVID‐19 survivors showed markedly poorer HRQOL compared with pre‐COVID‐19 times or controls (Dorri et al. 2021). Notably, persistent symptoms have a similar impact on individuals' HRQOL as the acute phase of infection (Sanchez‐Ramirez et al. 2021).

Findings regarding HRQOL among COVID‐19 survivors vary across studies due to differences in study populations, HRQOL measurement instruments, infection severity, follow‐up duration and assessed clinical outcomes (Sanchez‐Ramirez et al. 2021; Ma et al. 2022; de Oliveira Almeida et al. 2023). Although the greatest impact appears to occur among individuals with mild‐to‐moderate illness, most previous studies have primarily focused on hospitalized patients, which likely involved patients who experienced severe infection (de Oliveira Almeida et al. 2023). Furthermore, evidence regarding HRQOL beyond 1 year after infection remains limited, particularly among non‐hospitalized adults managed in primary care settings (Kim et al. 2023; Sanchez‐Ramirez et al. 2021). In addition, Long COVID research from low‐ and middle‐income countries remains scarce (Rajan et al. 2021; Sanchez‐Ramirez et al. 2021; de Oliveira Almeida et al. 2023).

The purpose of this study was to assess HRQOL among adults with Long COVID 2 years and beyond after acute COVID‐19 infection. The generic EQ‐5D‐5L instrument was used to measure HRQOL in adults diagnosed in primary care with mild‐to‐moderate infection during the 2021 pandemic.

## 2 Methods

### 2.1 Study Design

A cross‐sectional survey was conducted in Brazilian primary healthcare facilities between May 2023 and July 2024. Two instruments were administered to adults diagnosed with mild‐to‐moderate COVID‐19 between January and December 2021: (1) a standardized questionnaire comprising 18 validated clinical outcomes of Long COVID (Alverga 2023) and (2) the EQ‐5D‐5L generic instrument used to measure the health‐related quality of life (HRQOL).

The standardized questionnaire used in this study; the participants selection procedures, and Long COVID clinical outcomes have been described in a previously published article (Ferreira et al. 2025).

### 2.2 Settings

The study was conducted in six Family Health Care Units (Unidade de Saúde da Família—USF), in João Pessoa city, Paraiba State, Brazil. USFs are public ambulatory primary healthcare facilities distributed throughout all territories of Brazil as part of the National Primary Health Care Policy established by the Brazilian Ministry of Health (2017). Each USF is composed of a family health team consisting of physicians, nurses, nursing assistants and community health workers. Additional specialized services are provided by multidisciplinary teams that may include psychologists, social workers, pharmacists, speech and hearing therapists, gynaecologists/obstetricians, paediatricians, geriatricians and others according to local needs (Brazilian Ministry of Health 2017).

Each health team typically covers 2000 to 4000 individuals residing within a defined geographic area located as close as possible to the users. USFs provide continuous basic assistance organized for the enrolled population, with guaranteed access to diagnostic and laboratory support at the ambulatory level (Brazilian Ministry of Health 2008).

The six USFs included in this study were randomly selected from the 87 healthcare units operating in the city of João Pessoa (João Pessoa City Hall, Municipal Health Department 2024). The selected USFs varied in terms of the territories covered, population characteristics, and levels of socio‐environmental vulnerability. Four of the six USFs served territories with high or very high levels of socio‐environmental vulnerability (slums), characterized by elevated social vulnerability, environmental risk and degradation.

### 2.3 Ethical Considerations

The study protocol was approved by the Research Ethics Committee of the Center for Health Sciences of the Federal University of Paraiba (Comitê de Ética em Pesquisa do Centro de Ciências da Saúde da Universidade Federal da Paraíba—CCS/UFPB), as required for research involving human participants (n°5.376.051; CAAE: 55764722.4.0000.5188; approved on April 28, 2022).

The study did not involve therapeutic, clinical or diagnostic interventions and presented minimal risk to participants. Written informed consent was obtained from all participants before the interviews were conducted.

### 2.4 Long COVID Definition

The definition of Long COVID followed the World Health Organization (WHO) post‐COVID‐19 condition criteria (Soriano et al. 2022). According to the WHO definition, ‘post‐COVID‐19 condition occurs in individuals with a history of probable or confirmed SARS CoV‐2 infection, usually 3 months from the onset of COVID‐19 with symptoms and that last for at least 2 months and cannot be explained by an alternative diagnosis’.

Participants were asked whether any of the listed symptoms had occurred within 3 months after SARS‐CoV‐2 infection and how long the symptoms persisted. Symptom duration was categorized into four time points: < 1 month, 1–2 months, 2–3 months, and > 3 months. Cases were classified as Long COVID when symptoms developed within 3 months after the onset of infection, persisted for at least 2 months, and could not be explained by an alternative diagnosis.

### 2.5 Study Population

The study population consisted of adults who met the following eligibility criteria: age ≥ 18 years; a history of mild‐to moderate COVID‐19 illness diagnosed between January 1 and December 31, 2021; laboratory‐confirmed SARS‐CoV‐2 infection; and availability for follow‐up contact (i.e., participants with valid contact information who were permanent residents of João Pessoa).

Mild‐to‐moderate COVID‐19 diagnoses were established by physicians affiliated with the USFs. Mild COVID‐19 cases were defined as symptomatic patients without evidence of viral pneumonia or hypoxia. Moderate COVID‐19 cases included patients with clinical signs of non‐severe pneumonia who did not require hospitalization.

### 2.6 Sampling

A convenience sampling strategy was adopted. Participants were selected based on confirmed positive COVID‐19 test results and willingness to participate in the study. The sample consisted of all eligible individuals attending the selected USFs who had a confirmed SARS‐CoV‐2 infection and agreed to participate in the interviews.

### 2.7 Recruitment

**FIGURE 1:**
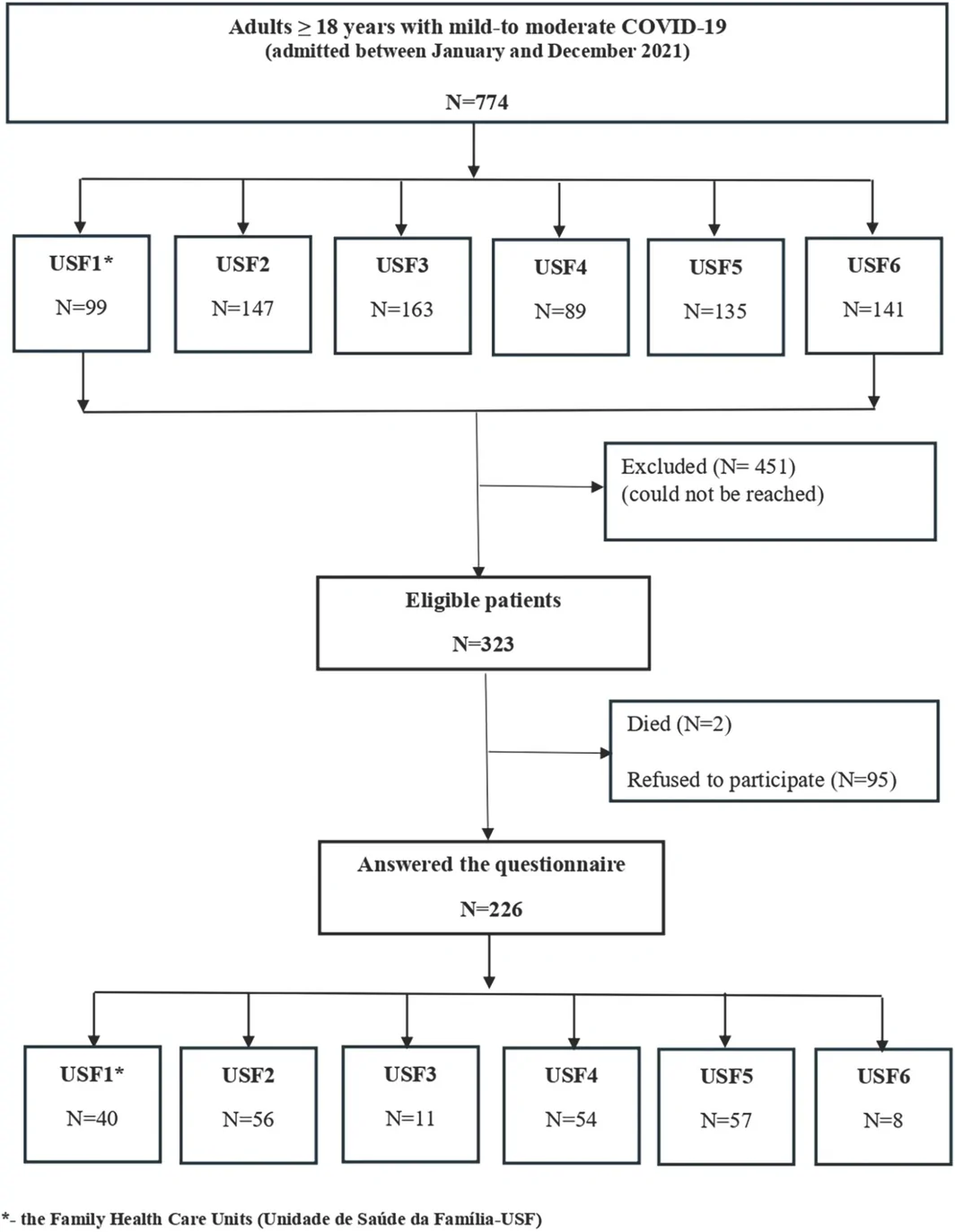
Participant selection flowchart. [Colour figure can be viewed at wileyonlinelibrary.com]

Lists of potentially eligible individuals, including COVID‐19 laboratory results and contact information, were obtained from USF medical records and electronic archives. Recruitment areas varied in population characteristics to ensure ethnic and sociodemographic diversity among study participants. Recruitment was conducted by trained interviewers. Potential participants were contacted through home visits or telephone calls and were invited to participate in the study. Figure 1 illustrates the participant selection process.

### 2.8 Data Collection and Data Management

Both instruments—the standardized Long COVID questionnaire and the generic EQ‐5D‐5L instrument—were administered at the same appointment to interview study participants.

All participants were interviewed in person, either during home visits or at the USF facilities, per their preference. Assistance was provided for participants who were unable to complete the questionnaires independently (e.g., due to illiteracy or difficulty writing). The average time to complete the survey was estimated as 30 min per interview. After completing the interview, the data were archived and transferred to a secure electronic database.

Each interviewer underwent a selection process and received training from a senior investigator experienced in conducting qualitative studies. Certain measures were adopted to minimize potential interview bias: (a) interviewer training sessions were conducted twice: at the beginning and midpoint of the survey; (b) all interviewers were instructed to strictly follow the interview guide and avoid any comments or behaviours that could influence participants' responses.

### 2.9 HRQOL Measures

The generic EuroQol 5‐dimension, 5‐level instrument (EQ‐5D‐5L) was used to assess the health‐related quality of life (HRQOL) of the study population (EuroQol Research Foundation 2025). The study used the officially translated and validated self‐administered Brazilian Portuguese version of the EQ‐5D‐5L provided by the EuroQol Group (version 1.2, 01.03.2023).

The EQ‐5D‐5L is a standardized instrument for assessing health‐related quality of life and has demonstrated satisfactory psychometric properties, including good validity, reliability, and sensitivity for measuring health outcomes across diverse clinical and population‐based settings (Feng et al. 2021). The Brazilian Portuguese version of the EQ‐5D‐5L has demonstrated adequate psychometric performance supporting its use for assessing health‐related quality of life in Portuguese‐speaking populations (Nakamura et al. 2022). The EQ‐5D‐5L has been recommended by an international Delphi consensus for measuring HRQOL in adults with Long COVID (Gorst et al. 2023).

The instrument comprises two principal components—a descriptive system and a visual analogue scale (EQ‐VAS) (Devlin et al. 2020). The descriptive system of five dimensions (mobility, self‐care, usual activities, pain/discomfort and anxiety/depression) is accompanied by five response levels (no problems, slight problems, moderate problems, severe problems and unable to/extreme problems). Participants were asked to indicate their health state by checking the most appropriate response level for each dimension. Responses were coded with a 5‐digit number representing distinct health states (e.g., 11,111—no problem in any dimension and 55,555—the extreme problems in all dimensions) (Devlin et al. 2020).

The EQ‐VAS assesses the individual's overall current health on a visual analogue scale ranging from 0 to 100, where the endpoints are labelled ‘The best health you can imagine’ and ‘The worst health you can imagine’. Participants were asked to indicate the number that best reflected their current perception of own health.

### 2.10 Statistical Analysis

All statistical analyses were performed using IBM SPSS Statistics, version 29.0.2.0.

#### 2.10.1 Descriptive Statistics

Demographic characteristics of the study population, race/ethnic background, educational level, COVID‐19 vaccination history, COVID‐19 related symptoms and the presence of comorbidities were drawn from a standardized questionnaire (Ferreira et al. 2025).

Continuous variables are presented as medians and interquartile ranges (IQR), whereas categorical variables are presented as absolute values with percentages (%). Comparisons between categorical variables were performed with the chi‐squared test or Fisher's exact test, as appropriate. The Mann–Whitney *U* test was used to compare continuous variables (age and EQ‐VAS) between two groups. Three missing values on education level were excluded from the analysis.

Statistical significance was defined as *p*‐value < 0.05. Effect sizes were calculated using rank‐biserial correlation (*r*) for continuous variables analysed with the Mann–Whitney U test, Cohen's *h* for differences in proportions, and Cramér's V for categorical variables with more than two categories.

#### 2.10.2 Analysis of Health‐Related Quality of Life (HRQOL)

Absolute numbers and proportions (%) were calculated for each response level of the EQ‐5D‐5L descriptive system, as well as for dichotomized variables with two categories (level 1 = no problem, levels 2 to 5 = any problems). Comparisons between categorical variables were performed using the chi‐squared test or Fisher's exact test, as appropriate. Effect sizes (Cramér's *V*) were calculated along with *p*‐values for each EQ‐5D‐5L dimension with five response levels (Appendix S1).

Participants' EQ‐5D‐5L responses were converted into utility scores. The Brazilian value set for EQ‐5D‐5L is yet to be developed; hence, the United States (US) value set to obtain utility scores was used instead (Pickard et al. 2019). The US index values range from 1 (full health) to 0 (death), with values below 0 (down to −0.109) representing health states considered ‘worse than death’. The area under the curve (AUC), 95% confidence intervals and standard errors under the nonparametric assumption were estimated. Receiver operating characteristic (ROC) curves for each binary EQ‐5D‐5L dimension are presented in Appendix S2. Overall model performance was considered good or excellent when AUC values exceeded 0.8 or 0.9, respectively, depending on the HRQOL dimension evaluated (Appendix S3).

Because utility scores were not normally distributed, medians with interquartile ranges (IQR) were calculated for explanatory variables. Comparisons between groups and sub‐groups were performed using the nonparametric Mann–Whitney U‐test and Kruskal–Wallis test, as appropriate. Statistical significance was defined as *p* < 0.05. Effect sizes were also calculated using *r* for the Mann–Whitney U test and epsilon‐squared (*ε* ^2^) for the Kruskal–Wallis test.

A multivariable logistic regression model was applied to investigate the association between Long COVID and EQ‐5D‐5L health dimensions. The outcome variable was Long COVID status classified as ‘yes’ and ‘no’. The primary explanatory variables included the EQ‐5D‐5L dimensions (mobility, self‐care, usual activities, pain/discomfort and anxiety/depression), each categorized as the presence of any issues versus no issues.

Univariate logistic regression models were used to estimate unadjusted odds ratios (OR) and corresponding 95% confidence intervals for the association between each EQ‐5D‐5L dimension and Long COVID. Multivariate logistic regression analysis was subsequently performed to estimate adjusted odds ratios (aOR) while controlling for selected covariates.

Covariates included gender (female as the reference category), age group (age 18–29 years as the reference category), comorbidity status (no comorbidities as the reference category), race (White participants as the reference category) and years of schooling (< 12 years as the reference category). A stepwise regression approach was used to select covariates associated at *p* < 0.20. Gender and comorbidity status were retained in the final model regardless of statistical significance.

The final model demonstrated an overall classification accuracy of 82.7%, correctly identifying 141 positive cases (true positives) and 46 negative cases (true negatives).

## 3 Results

### 3.1 Baseline Characteristics

Table 1 summarizes the baseline characteristics of the study population. A total of 226 individuals with a history of mild‐to‐moderate SARS‐CoV‐2 infection responded to the questionnaire. The median age was 49.0 years (IQR 37.8–62.0), and 68.1% were female. Among the participants, 73.0% were non‐White, 71.7% had fewer than 12 years of schooling, and 36.7% reported at least one pre‐existing chronic condition. The most common conditions were arterial hypertension (25.2%) and diabetes (11.1%). The proportion of those who received COVID‐19 vaccination at primary care was 98.7%.

**TABLE 1:** Baseline characteristics of the participants.

Variables	All	With long COVID	Without long COVID	*p* *	Effect size^c^
	*N* = 226	*N* = 156	*N* = 70		
	*n* (%)	*n* (%)	*n* (%)		
Total	226 (100)	156 (69.0)	70 (31.0)	—	—
Sex					
Males	72 (31.9)	42 (26.9)	30 (42.9)	0.017	−0.33
Females	154 (68.1)	114 (73.1)	40 (57.1)		
Age (years), Median (IQR)	49.0 (37.8–62.0)	49.5 (40.0–63.8)	42.5 (34.0–60.0)	0.032	−0.14
< 60	159 (70.4)	107 (68.6)	52 (74.3)	0.386	−0.13
≥ 60	67 (29.6)	49 (31.4)	18 (25.7)		
18–29	22 (9.7)	11 (7.1)	11 (15.7)	0.081	0.16
30–39	44 (19.5)	27 (17.3)	17 (24.3)		
40–49	50 (22.1)	40 (25.6)	10 (14.3)		
50–59	43 (19.1)	29 (18.6)	14 (20.0)		
60–69	40 (17.7)	27 (17.3)	13 (18.6)		
70+	27 (11.9)	22 (14.1)	5 (7.1)		
Race/Ethnicity					
White	61 (27.0)	42 (26.9)	19 (27.1)	0.973	−0.01
Non‐White	165 (73.0)	114 (73.1)	51 (72.9)		
Education^a^ (years)					
< 12	162 (71.7)	107 (68.6)	55 (78.6)	0.113	−0.23
≥ 12	61 (27.0)	47 (30.1)	14 (20.0)		
Missing values	3 (1.3)	2 (1.3)	1 (1.4)		
Symptoms					
None	16 (7.1)	—	16 (22.9)	< 0.001	0.99
Any	210 (92.9)	156 (100)	54 (77.1)		
< 5	78 (34.5)	28 (17.9)	50 (71.4)	< 0.001	1.15
≥ 5	148 (65.5)	128 (82.1)	20 (28.6)		
Comorbidities					
None	143 (63.3)	94 (60.3)	49 (70.0)	0.483	0.20
Any	83 (36.7)	62 (39.7)	21 (30.0)		
AH	57 (25.2)	43 (27.6)	14 (20.0)	0.226	0.18
Diabetes	25 (11.1)	17 (10.9)	8 (11.4)	0.906	−0.02
MD	11 (4.9)	9 (5.8)	2 (2.9)	0.347	0.16
Respiratory diseases	4 (1.8)	4 (2.6)	—	0.176	0.23
Cardiac diseases	3 (1.3)	1 (0.6)	2 (2.9)	0.177	−0.21
Other^b^	18 (8.0)	16 (10.3)	2 (2.9)	0.026	0.38
COVID‐19 vaccination					
None	3 (1.3)	2 (1.3)	1 (1.4)	0.928	0.01
≥ 1	223 (98.7)	154 (98.7)	69 (98.6)		
EQ‐5D‐5L					
EQ‐VAS, Median (IQR)	70 (60–81)	70 (50–80)	80 (70–90)	< 0.001	−0.36

Abbreviations: AH, arterial hypertension; MD, musculoskeletal diseases; SD, standard deviation.

^a^ Three missing values.

^b^ Category ‘Other Comorbidities’ includes anaemia, asthma, allergy, chronic sinusitis, thyroid diseases and eye conditions.

^c^ Effect sizes are reported as *r* for continuous variables based on the Mann–Whitney U test, Cohen's *h* for differences in proportions, and Cramér's V for categorical variables with more than two categories.

* Statistical significance set at *p* < 0.05.

Overall, 69.0% (156/226) of participants met the definition of Long COVID, reporting at least one persistent symptom 3 months and beyond after COVID‐19 illness. Compared with those without Long COVID, adults in this group were older (median 49.5 vs. 42.5 years, *r* = −0.14, *p* = 0.032) and more likely to be female (73.1% vs. 57.1%, Cohen's *h* = −0.33, *p* = 0.017). No statistically significant differences were observed for other demographic variables (Table 1).

Symptom burden differed markedly between the two groups. All individuals with Long COVID were symptomatic, with a significantly greater proportion of those who reported any symptom (100% vs. 77.1%) and ≥ 5 symptoms (82.1% vs. 28.6%) compared with those without Long COVID. Those differences were large in magnitude (Cohen's *h* = 0.99 and 1.15, respectively; both *p* < 0.001).

### 3.2 Health‐Related Quality of Life (HRQOL)

**FIGURE 2:**
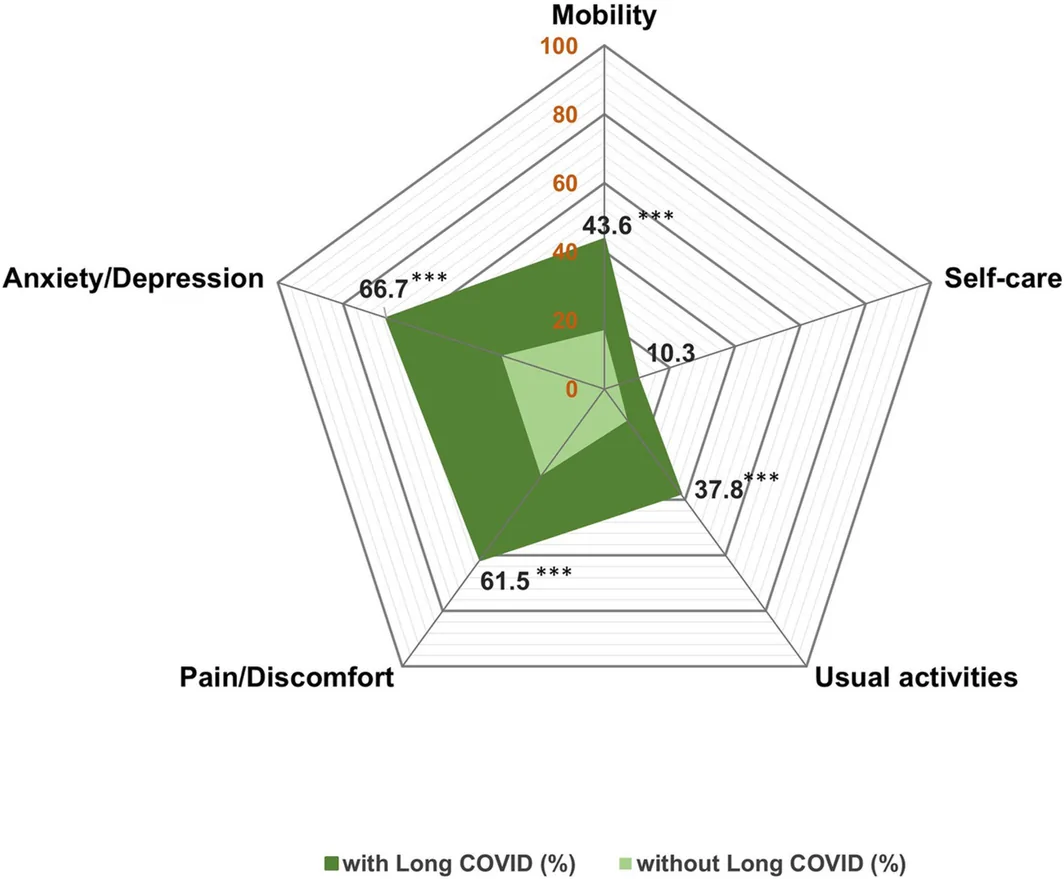
Proportions (%) of respondents reporting any health issues, plotted separately for individuals with and without Long COVID. ***p < 0.001. [Colour figure can be viewed at wileyonlinelibrary.com]

Individuals with Long COVID reported a higher prevalence of health issues across four of five EQ‐5D‐5L dimensions compared with those without persistent illness (Figure 2). The largest differences were observed for anxiety/depression (66.7% vs. 31.4%, *p* < 0.001), followed by pain/discomfort (61.5% vs. 31.4%, *p* < 0.001), mobility (43.6% vs. 17.1%, *p* < 0.001) and usual activities (37.8% vs. 11.4%, *p* < 0.001). No statistically significant difference in proportions was found for the self‐care dimension (10.3% vs. 4.3%, *p* = 0.135).

Among individuals with Long COVID, the most common observed health issues were slight or moderate (Appendix S1). Moderate problems were most frequently reported for pain/discomfort (32.1%), followed by anxiety/depression (25.0%) and mobility (15.4%). The highest proportion of respondents with severe and extreme health issues was identified for anxiety/depression (12.2% and 2.6%, respectively). Despite these differences, a substantial proportion of respondents—particularly in the self‐care and mobility dimensions—reported no problems. Effect sizes indicated small‐to‐moderate differences between two groups across EQ‐5D‐5L dimensions (Cramér's *V* = 0.27–0.31; Appendix S1).

Comparisons between participants with and without Long COVID showed consistently lower medians of utility scores in the Long COVID group across all sub‐categories, including sex, age, ethnicity, education and comorbidities (Table 2). Compared with those without Long COVID, adults with Long COVID had significantly lower medians of utility scores regardless of sex, age, ethnicity, education and comorbidities. The largest differences between two groups were observed for comorbidities (0.730 vs. 0.943, *p* < 0.001, *r* = −0.61), females (0.747 vs. 0.972, *r* = −0.53), individuals aged 18–29 years (0.877 vs. 1.0, *p* = 0.007, *r* = −0.57), those aged 60–69 years (0.738 vs. 0.943, *p* = 0.001, *r* = −0.50), participants with less than 12 years of schooling (0.770 vs. 1.0, *p* < 0.001, *r* = −0.48) and non‐White individuals (0.780 vs. 1.0, *p* < 0.001, *r* = −0.47).

**TABLE 2:** EQ‐5D‐5L utility scores comparing adults with versus without Long COVID.

Variables	With long COVID				Without long COVID				*p* ^b^	Effect size^c^
	*N* = 156				*N* = 70					
	Median	IQR	*p* *	Effect size^c^	Median	IQR	*p* *	Effect size^c^		
Overall utility score	0.784	0.633–0.902	—	—	1.000	0.877–1.000	—	—	< 0.001	−0.45
Sex										
Male	0.902	0.734–0.957	< 0.001	−0.31	1.000	0.876–1.000	0.985	0.00	0.027	−0.26
Female	0.747	0.604–0.877			0.972	0.879–1.000			< 0.001	−0.53
Symptoms										
None	—	—	NA	NA	1.000	1.000–1.000	0.013	−0.30	NA	NA
Any	0.784	0.633–0.902			0.943	0.865–1.000			< 0.001	−0.38
< 5	0.942	0.838–1.000	< 0.001	−0.35	1.000	0.942–1.000	< 0.001	−0.42	0.023	−0.26
≥ 5	0.749	0.603–0.882			0.880	0.721–0.986			0.012	−0.21
Age (years)										
< 60	0.817	0.641–0.940	0.185	−0.11	1.000	0.887–1.000	0.427	−0.09	< 0.001	−0.46
≥ 60	0.738	0.610–0.862			0.943	0.830–1.000			< 0.001	−0.45
18–29	0.877	0.776–0.943	0.033	0.05	1.000	0.940–1.000	0.528	0.00	0.007	−0.57
30–39	0.875	0.701–0.940			1.000	0.921–1.000			0.002	−0.46
40–49	0.831	0.629–0.943			1.000	0.812–1.000			0.039	−0.29
50–59	0.695	0.554–0.875			0.972	0.804–1.000			0.001	−0.49
60–69	0.738	0.602–0.847			0.943	0.816–1.000			0.001	−0.50
70+	0.731	0.624–0.950			0.953	0.894–1.000			0.028	−0.43
Race										
White	0.844	0.619–0.943	0.627	−0.04	0.944	0.844–1.000	0.662	−0.05	0.002	−0.39
Non‐White	0.780	0.641–0.902			1.000	0.883–1.000			< 0.001	−0.47
Education^a^ (years)										
< 12	0.770	0.630–0.902	0.452	−0.06	1.000	0.883–1.000	0.325	−0.12	< 0.001	−0.48
≥ 12	0.844	0.648–0.940			0.943	0.865–1.000			0.009	−0.33
Comorbidities										
None	0.817	0.695–0.941	0.019	−0.19	1.000	0.921–1.000	0.156	−0.17	< 0.001	−0.30
Any	0.730	0.585–0.879			0.943	0.802–1.000			< 0.001	−0.61

Abbreviations: IQR, interquartile range; NA, not applicable.

^a^ Three missing values.

^b^ Statistical differences between groups (Long COVID vs. without Long COVID); statistical significance set at *p* < 0.05.

^c^ Effect sizes are reported as *r* for Mann–Whitney U tests and epsilon‐squared (*ε* ^2^) for Kruskal–Wallis tests.

* Statistical differences within groups; statistical significance set at *p* < 0.05.

Among participants with Long COVID, median utility scores were lower in women than in men (0.747 vs. 0.902, *p* < 0.001, *r* = −0.31), and in those reporting ≥ 5 symptoms compared with those reporting < 5 symptoms (0.749 vs. 0.942, *p* < 0.001, *r* = −0.35). Participants with comorbidities also had lower scores than those without pre‐existing chronic conditions (0.730 vs. 0.817, *p* = 0.019, *r* = −0.19), although the difference was smaller.

Across age groups, utility scores varied, with lower median values observed in older participants. The lowest median utility score was found among those aged 50–59 years (0.695, IQR = 0.554–0.875).

The results of the EQ‐VAS analysis showed significantly lower median scores among adults with Long COVID compared with those without Long COVID (70 vs. 80, *p* < 0.001, *r* = −0.36; Table 1).

**FIGURE 3:**
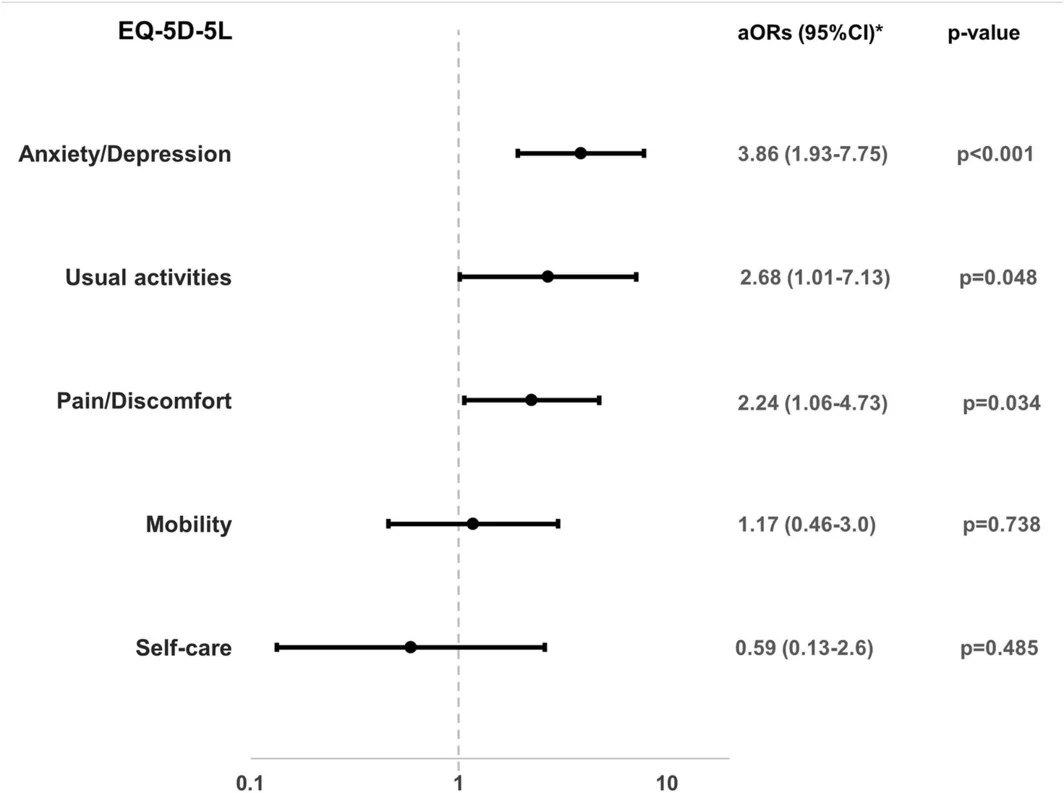
Adjusted odds ratios for EQ‐5D‐5L dimensions with a predicted probability of having Long COVID. AOR, adjusted odds ratio; *adjusted for sex, age, and comorbidities.

Figure 3 shows the estimated adjusted odds ratios (aOR) with the respective 95% confidence intervals for five EQ‐5D‐5L dimensions after applying multivariate logistic regression models. After adjustment for age, sex, and comorbidities, participants with Long COVID had higher odds of reporting issues with anxiety/depression (aOR: 3.86, 95% CI: 1.93–7.75, *p* < 0.001), pain/discomfort (aOR: 2.24, 95% CI: 1.06–4.73, *p* = 0.034), and usual activities (aOR: 2.68, 95% CI: 1.01–7.13, *p* = 0.048) as compared with those without Long COVID (Figure 3, Appendix S1).

## 4 Discussion

More than two‐thirds of adults with Long COVID in our study reported continuous health issues 2 years and beyond after non‐severe COVID‐19 illness. This finding is particularly notable given that the study population had a median age of 49 years, all participants experienced non‐severe infection, and all received medical care in primary care settings during or after the acute phase of infection. Furthermore, this study is among the few from a middle‐income country and from primary care settings to report persistent health problems following non‐severe COVID‐19 infection.

Long COVID was more frequently reported among women, non‐White individuals, adults with lower educational attainment and residents of territories characterized by high or very high socio‐environmental vulnerability. All individuals with Long COVID reported symptoms, with a significantly higher prevalence of both individual symptoms and multiple concurrent symptoms compared with adults without Long COVID. More than one‐third of individuals with Long COVID also had pre‐existing chronic conditions.

Adults with Long COVID demonstrated poorer health outcomes than those without persistent illness, regardless of age, sex, ethnicity, education and pre‐existing chronic conditions. The most common health issues reported among respondents with Long COVID were anxiety or depression, pain or discomfort, problems with mobility and reduced ability to perform usual activities. Furthermore, three of five EQ‐5D‐5L dimensions—anxiety/depression, pain/discomfort and usual activities—were significantly associated with Long COVID.

The results of our study strengthen previous evidence demonstrating reduced health‐related quality of life (HRQOL) among individuals experiencing long‐lasting effects following COVID‐19 infection (Sanchez‐Ramirez et al. 2021; Ma et al. 2022; Dorri et al. 2021; Kim et al. 2023; de Oliveira Almeida et al. 2023). In a meta‐analysis of seven studies reporting HRQOL outcomes among 24 cohort studies, 52% of adults with persistent symptoms had decreased HRQOL (Sanchez‐Ramirez et al. 2021). Although some studies included in this meta‐analysis reported larger declines in HRQOL among individuals who experienced severe illness (Huang et al. 2021; Walle‐Hansen et al. 2021), other studies showed substantially poorer health outcomes among adults with mild infection (van den Borst et al. 2021).

The high proportion of adults in our study reporting continuing health problems 2 years and beyond after non‐severe infection is particularly concerning. Similarly, Kim et al. (2023), in a prospective online survey using EQ‐5D‐5L measures among adults with persistent symptoms, demonstrated that HRQOL remained affected 2 years after acute infection. Furthermore, in this study, EQ‐5D‐5L scores at both 12 and at 24 months after COVID‐19 showed a distribution similar to that observed in our study (Kim et al. 2023). The proportion of participants reporting slight problems across the five EQ‐5D‐5L dimensions was also comparable to our findings, although a higher proportion of moderate‐to‐severe health issues was observed among respondents in our study. However, the online survey did not include adults aged over 70 years, and those who could not fill in online forms; hence, it may impact individuals' health outcomes differently.

In our study, anxiety/depression was the most frequently reported health problems among adults with Long COVID, followed by pain/discomfort, mobility limitations and difficulties performing. However, no statistically significant difference between two groups was identified for self‐care problems. A substantial proportion of participants with Long COVID reported a variety of symptoms, including fatigue, anxiety, muscle and joint pain, and post‐exertional malaise, all of which may contribute to poorer HRQOL outcomes (Ferreira et al. 2025). Similar findings were reported in a recent meta‐analysis, in which pain/discomfort and anxiety/depression were the most common health problems among adults with persistent symptoms 1 year after acute infection (Ma et al. 2022). Consistent with our findings, self‐care was not reported as a common health problem among individuals with Long COVID (Ma et al. 2022).

More than two‐thirds of adults in our study reported anxiety or depression, both of which were strongly associated with COVID‐19 long‐lasting effects. Fatigue, pain, post‐exertional malaise, difficulties with executive functioning and memory issues were among the most common neurological features in our study (Ferreira et al. 2025). These symptoms may contribute to mobility limitations and reduced ability to perform daily activities.

A meta‐analysis examining HRQOL and psychological problems among COVID‐19 survivors demonstrated that anxiety and depression during the COVID‐19 pandemic were strongly associated with decreased HRQOL (Dorri et al. 2021). Similarly, the results of an international cohort study involving 3762 individuals with Long COVID confirmed that the presence of neuropsychiatric symptoms, particularly fatigue, cognitive disfunctions and executive functioning problems, may severely impact the individual's daily functioning and their ability to go back to work (Davis et al. 2021). In addition, two systematic reviews reported declines in daily activities of those with Long COVID with decreasing HRQOL, regardless of the scales applied (de Oliveira Almeida et al. 2023; Pizarro‐Pennarolli et al. 2021).

Among respondents with Long COVID in our study, women, older adults, non‐White individuals, adults with pre‐existing chronic diseases and those with fewer years of schooling all had significantly poorer HRQOL. Furthermore, symptomatic adults reporting a greater number of COVID‐related symptoms had significantly poorer HRQOL outcomes than those with fewer than five symptoms.

Women appear to be at greater risk of developing Long COVID and experiencing poorer quality of life outcomes compared to men, although the mechanisms underlying this increased susceptibility remain unclear (Sanchez‐Ramirez et al. 2021; Davis et al. 2021; Ma et al. 2022; Luo et al. 2024). In addition, women appear to be more susceptible than men to post‐COVID‐19 sequelae involving psychiatric and neurological impairments, including anxiety, depression and cognitive disorders (Davis et al. 2021), all of which were highly prevalent in our study.

Adults aged ≥ 60 years had the highest prevalence of comorbidities in our study, including diabetes and hypertension; hence, this may partly explain the poorer health outcomes compared to other age groups. In addition, SARS‐CoV‐2 infection may exacerbate existing chronic conditions and, together with less robust immune responses among older adults, may increase susceptibility to chronic inflammation (Mansell et al. 2022). A longitudinal study of Long COVID during two epidemic peaks in Brazil described a correlation between pre‐existing chronic conditions and the persistence of COVID‐19‐related symptoms (de Miranda et al. 2022).

Health inequities may place racial and ethnic minority populations at greater risk for developing Long COVID (Khullar et al. 2023; Xiang et al. 2025). A retrospective analysis of 62,339 electronic health records from individuals with confirmed COVID‐19 suggested that racial and ethnic minority groups may experience a greater overall burden of potential Long COVID symptoms compared with White individuals (Khullar et al. 2023). There remains a research gap regarding the effect of social disadvantages—including racial and ethnic minority status, lower education, and poor living conditions—on Long COVID development and related health outcomes (Xiang et al. 2025). A few studies have suggested that individuals with lower socioeconomic status and disadvantaged living environments may face a higher risk of Long COVID (Berger et al. 2021). However, HRQOL among socially vulnerable populations with Long COVID remains insufficiently explored.

There are a few studies from Brazil that have previously described HRQOL among adults with Long COVID (de Azevedo Vieira et al. 2023; Azambuja et al. 2024; de Oliveira et al. 2022; D'Ávila et al. 2023; Prata et al. 2024; da Silveira et al. 2024), although evidence in this field is still evolving. We identified two Brazilian studies that assessed HRQOL using the EQ‐5D‐5L instrument. One study explored the impact of Long COVID in a socially vulnerable community (Azambuja et al. 2024), whereas the second investigated persistent symptoms among healthcare workers (D'Ávila et al. 2023). Neither study provided utility score estimates, which prevents us from comparing the results.

The study of a Brazilian vulnerable community reported predominantly slight‐to‐moderate problems related to anxiety/depression, pain/discomfort, mobility and usual activities, which are in keeping with our findings. Although HRQOL in the study by Azambuja et al. (2024) was assessed primarily among previously hospitalized patients (99.8%), our study demonstrated poorer HRQOL outcomes among individuals experiencing persistent symptoms after mild‐to‐moderate COVID‐19. Similarly, a previous study evaluating long‐lasting effects of COVID‐19 reported poorer persistent health outcomes among patients with mild illness compared with previously hospitalized individuals (van den Borst et al. 2021).

### 4.1 Strengths and Limitations

To our knowledge, this is the first study from Brazil to assess the HRQOL among adults with persistent Long COVID symptoms in primary care settings more than 2 years after the COVID‐19 pandemic. We used a standardized questionnaire with a set of predefined and validated clinical outcomes, which reduced potential heterogeneity in self‐reported symptoms. In addition, we used the EQ‐5D‐5L instrument, which has been recommended by an international Delphi consensus for measuring HRQOL among adults with Long COVID in both research and clinical practice. Furthermore, face‐to‐face interviews enabled a more comprehensive assessment of the lived experiences of COVID‐19 survivors.

This study has several limitations, and the findings should therefore be interpreted cautiously. First, the US population value set was used to estimate EQ‐5D‐5L utility scores; thus, it may not accurately represent the Brazilian population's values. Second, the study used convenience sampling and included a relatively small sample size, which may limit generalizability and statistical power. Third, COVID‐19‐related symptoms were assessed retrospectively, potentially introducing recall bias.

Although most adults in this study were vaccinated within the universal Brazilian COVID‐19 vaccination programme, we were unable to determine whether vaccination occurred before or after infection. Furthermore, more women responded to our questionnaire; thus, it may influence the results. Finally, testing capacity for SARS‐CoV‐2 infection within Brazilian primary care settings during the 2021 pandemic was limited; consequently, some infected individuals may not have been tested, and not all positive test results may have been routinely recorded.

### 4.2 Implications for Policy and Practice

This study provides important insights into the lived experiences of COVID‐19 survivors more than 2 years after acute infection, particularly within primary care settings. The findings highlight persistent and multidimensional health problems experienced after mild‐to‐moderate COVID‐19 illness, including physical limitations, psychological distress, and difficulties performing daily activities.

The observed reduction in HRQOL among adults following non‐severe infection, particularly among socially vulnerable populations, has important implications for healthcare planning and resource allocation. The findings indicate a need for multidisciplinary rehabilitation programmes within primary care settings tailored to the needs of COVID‐19 survivors and may help inform future healthcare planning.

Furthermore, the high prevalence of persistent psychological symptoms highlights the importance of integrating mental health screening and interventions into standard models of care. This study also contributes to the growing recognition of HRQOL as a critical component in the evaluation and management of Long COVID. The findings emphasize the importance of incorporating patient‐reported quality of life outcomes into both healthcare policy and clinical practice, particularly within primary care systems, to better address the complex and long‐term impacts of Long COVID.

## 5 Conclusions

COVID‐19 survivors with persistent symptoms lasting 2 years or longer after non‐severe infection experienced a substantial decline in health‐related quality of life (HRQOL). Adults with Long COVID reported significantly poorer HRQOL outcomes than those without Long COVID, regardless of sex, age, ethnicity, education level or comorbidities. These findings support the implementation of targeted interventions and rehabilitation services in primary care for individuals experiencing long‐term health problems following COVID‐19 illness.

## Author Contributions

All authors were responsible for conceptualization, study design, analytic approach and data curation. A.M.S.F. and J.A.d.N. performed statistical analyses. A.M.S.F. wrote the original manuscript draft. All authors contributed to critically revising and approved the final version of the manuscript for publication. The corresponding author (A.M.S.F.) attests that all listed authors meet authorship criteria and that no others meeting the criteria have been omitted. There is a statistician (J.A.d.N.) on the author team.

## Funding

The study was funded by the Fundação de Apoio à Pesquisa do Estado da Paraíba (FAPESQ) (47449.673.29754.11082021), Brazil. The Article Processing Charge for the publication of this research was funded by the Coordenação de Aperfeiçoamento de Pessoal de Nível Superior (CAPES), Brazil.

## Conflicts of Interest

The authors declare no conflicts of interest.

## Supporting information


**Appendix S1:** Absolute frequencies and proportions (%) of respondents reporting problems in each EQ‐5D‐5L dimension.
**Appendix S2:** Receiver operating characteristic (ROC) analysis of the performance of the US value set for binary EQ‐5D‐5L dimensions.
**Appendix S3:** Receiver operating characteristic (ROC) analysis outcomes for the US value set.
**Appendix S4:** Crude and adjusted odds ratios for EQ‐5D‐5L dimensions by Long COVID status.

**Data S1:** STROBE Statement—Checklist of items that should be included in reports of cross‐sectional studies.

## Data Availability

The data that support the findings of this study are available on request from the corresponding author. The data are not publicly available due to privacy or ethical restrictions.
