# Supplementary material for: Health‐Related Quality of Life of Adults With Long COVID: A Cross‐Sectional Study in Primary Care
Source: J Clin Nurs. 2026 May 30;35(10):4365–77. doi: 10.1111/jocn.70383 (PMC13569344; doi:10.1111/jocn.70383)
Supplement: Supplementary file 1 [file JOCN-35-4365-s001.docx]

STROBE Statement—Checklist of items that should be included in reports of cross-sectional studies.

|  | **Item**  **No** | **Recommendation** | **Page**  **No** | **Relevant text from manuscript/Comments** |
| --- | --- | --- | --- | --- |
| **Title and abstract** | 1 | (a) Indicate the study’s design with a commonly used term in the title or the abstract | 2 | Cross-sectional study |
|  |  | (b) Provide in the abstract an informative and balanced summary of what was done and what was found | 2 | Health status was assessed using the EQ-5D-5L instrument in 226 adults diagnosed in primary care with mild-to-moderate COVID-19 during the 2021 pandemic. Data were collected through a cross-sectional survey using a standardized questionnaire with a set of validated clinical outcomes for Long COVID. Health utility scores were compared between adults with and without Long COVID. Multivariate logistic regressions were applied to investigate the relationship between Long COVID and health-related quality of life outcomes.  Adults with Long COVID had statistically significantly lower median utility scores than those without persistent symptoms. Poorer HRQOL was more evident among women, older adults, non-White individuals, participants with pre-existing chronic diseases, and those with lower educational attainment. Long COVID was associated with impairments in anxiety/depression, pain/discomfort, and usual activities. |
| **Introduction** |  |  |  |  |
| Background/rationale | 2 | Explain the scientific background and rationale for the investigation being reported | 4 | Evidence regarding HRQOL beyond one year after infection remains limited, particularly among non-hospitalized adults managed in primary care settings . In addition, Long COVID research from low- and middle-income countries remains scarce |
| Objectives | 3 | State specific objectives, including any prespecified hypotheses | 5 | The purpose of this study was to assess HRQOL among adults with Long COVID two years and beyond after acute COVID-19 infection. The generic EQ-5D-5L instrument was used to measure HRQOL in adults diagnosed in primary care with mild-to-moderate infection during the 2021 pandemic. |
| **Methods** |  |  |  |  |
| Study design | 4 | Present key elements of study design early in the paper | 5 | Cross-sectional survey. |
| Setting | 5 | Describe the setting, locations, and relevant dates, including periods of recruitment, exposure, follow-up, and data collection | 5, 7 | A cross-sectional survey was conducted in Brazilian primary healthcare facilities between May 2023 and July 2024. Two instruments were administered to adults diagnosed with mild-to-moderate COVID-19 between January and December 2021.  Both instruments - the standardized Long COVID questionnaire and the generic EQ-5D-5L instrument -were administered at the same appointment to interview study participants.  All participants were interviewed in person, either during home visits or at the USF facilities, per their preference. |
| Participants | 6 | (*a*) Give the eligibility criteria, and the sources and methods of selection of participants | 6,7 | The study population consisted of adults who met the following eligibility criteria: age ≥ 18 years; a history of mild-to moderate COVID-19 illness diagnosed between January 1 and December 31, 2021; laboratory-confirmed SARS-CoV-2 infection; and availability for follow-up contact (i.e., participants with valid contact information who were permanent residents of João Pessoa). |
| Variables | 7 | Clearly define all outcomes, exposures, predictors, potential confounders, and effect modifiers. Give diagnostic criteria, if applicable | 10 | The outcome variable was Long COVID status classified as “yes” and “no”. The primary explanatory variables included the EQ-5D-5L dimensions (mobility, self-care, usual activities, pain/discomfort and anxiety/depression), each categorized as the presence of any issues versus no issues. Covariates included gender (female as the reference category), age group (age 18-29 years as the reference category), comorbidity status (no comorbidities as the reference category), race (White participants as the-reference category), and years of schooling (<12 years as the reference category). A stepwise regression approach was used to select covariates associated at p<0.20. Gender and comorbidity status were retained in the final model regardless of statistical significance. |
| Data sources/ measurement | 8* | For each variable of interest, give sources of data and details of methods of assessment (measurement). Describe comparability of assessment methods if there is more than one group | 8 | The generic EuroQol 5-dimension, 5-level instrument (EQ-5D-5L) was used to access the health-related quality of life (HRQOL) of the study population. Participants were asked to indicate their health state by checking the most appropriate response level for each EQ-5D-5L dimension. |
| Bias | 9 | Describe any efforts to address potential sources of bias | 5,8 | The study used a standardized questionnaire with a set of validated Long COVID outcomes to overcome potential heterogeneity of findings.  Certain measures were adopted to minimize potential interview bias: (a) interviewer training sessions were conducted twice: at the beginning and midpoint of the survey; (b) all interviewers were instructed to strictly follow the interview guide and avoid any comments or behaviors that could influence participants’ responses. |
| Study size | 10 | Explain how the study size was arrived at | 7 | A convenience sampling strategy was adopted. Participant were selected based on confirmed positive COVID-19 test results and willingness to participate in the study. The sample consisted of all eligible attending the selected USFs who had a confirmed SARS-CoV-2 infection and agreed to participate in the interviews. |
| Quantitative variables | 11 | Explain how quantitative variables were handled in the analyses. If applicable, describe which groupings were chosen and why | 9 | Quantitative variables were initially assessed for distributional characteristics using descriptive statistics and normality tests. Continuous variables were summarized as medians and interquartile ranges, as appropriate. For inferential analyses, non-parametric tests were applied. |
| Statistical methods | 12 | (*a*) Describe all statistical methods, including those used to control for confounding | 9,10 | “Statistical analysis” section |
|  |  | (*b*) Describe any methods used to examine subgroups and interactions | 9,10 | Comparisons between categorical variables were performed with the chi-squared test or Fisher’s exact test, as appropriate. The Mann-Whitney U test was used to compare continuous variables (age and EQ-VAS) between two groups.  Because utility scores were not normally distributed, medians with interquartile ranges (IQR) were calculated for explanatory variables. Comparisons between groups and sub-groups were performed using the nonparametric Mann-Whitney U-test and Kruskal-Wallis, as appropriate. |
|  |  | (*c*) Explain how missing data were addressed | 9 | Three missing values on education level were excluded from the analysis. |
|  |  | (*d*) If applicable, describe analytical methods taking account of sampling strategy | 7 | A convenience sampling strategy was adopted. Participant were selected based on confirmed positive COVID-19 test results and willingness to participate in the study. The sample consisted of all eligible attending the selected USFs who had a confirmed SARS-CoV-2 infection and agreed to participate in the interviews. |
|  |  | (*e*) Describe any sensitivity analyses | 9 | The area under the curve (AUC), 95% confidence intervals, and standard errors under the nonparametric assumption were estimated. Overall model performance was considered good or excellent when AUC values exceeded 0.8 or 0.9, respectively, depending on the HRQOL dimension evaluated. |
| **Results** |  |  |  |  |
| Participants | 13* | (a) Report numbers of individuals at each stage of study—eg numbers potentially eligible, examined for eligibility, confirmed eligible, included in the study, completing follow-up, and analysed | - | The selection procedure is presented in Figure 1 |
|  |  | (b) Give reasons for non-participation at each stage | - | Figure 1 |
|  |  | (c) Consider use of a flow diagram | - | Figure 1 |
| Descriptive data | 14* | (a) Give characteristics of study participants (eg demographic, clinical, social) and information on exposures and potential confounders | - | Table 1- Baseline characteristics of study participants |
|  |  | (b) Indicate number of participants with missing data for each variable of interest |  | Table 1-Baseline characteristics of study participant. |
| Outcome data | 15* | Report numbers of outcome events or summary measures | - | Table 2- EQ-5D-5L utility scores comparing adults with versus without Long COVID |
| Main results | 16 | (*a*) Give unadjusted estimates and, if applicable, confounder-adjusted estimates and their precision (eg, 95% confidence interval). Make clear which confounders were adjusted for and why they were included | - | Appendix-Table 3 Crude and adjusted odds ratios for EQ-5D-5L dimensions by Long COVID status. |
|  |  | (*b*) Report category boundaries when continuous variables were categorized |  | Tables 1 and 2. |
|  |  | (*c*) If relevant, consider translating estimates of relative risk into absolute risk for a meaningful time period | N/A |  |
| Other analyses | 17 | Report other analyses done—eg analyses of subgroups and interactions, and sensitivity analyses | - | Supplementary materials |
| **Discussion** |  |  |  |  |
| Key results | 18 | Summarise key results with reference to study objectives | 12-14 | “Discussion” section |
| Limitations | 19 | Discuss limitations of the study, taking into account sources of potential bias or imprecision. Discuss both direction and magnitude of any potential bias | 16 | “Discussion” section; Strengths and limitations |
| Interpretation | 20 | Give a cautious overall interpretation of results considering objectives, limitations, multiplicity of analyses, results from similar studies, and other relevant evidence | 13-16 | “Discussion” section. |
| Generalisability | 21 | Discuss the generalisability (external validity) of the study results | 17 | “Discussion” section; Implications for Policy and Practice |
| **Other information** |  |  |  |  |
| Funding | 22 | Give the source of funding and the role of the funders for the present study and, if applicable, for the original study on which the present article is based |  | Title page |

*Give information separately for exposed and unexposed groups.

Note: An Explanation and Elaboration article discusses each checklist item and gives methodological background and published examples of transparent reporting. The STROBE checklist is best used in conjunction with this article (freely available on the Web sites of PLoS Medicine at http://www.plosmedicine.org/, Annals of Internal Medicine at http://www.annals.org/, and Epidemiology at http://www.epidem.com/). Information on the STROBE Initiative is available at www.strobe-statement.org.
