# Supplementary material for: Health‐Related Quality of Life of Adults With Long COVID: A Cross‐Sectional Study in Primary Care
Source: J Clin Nurs. 2026 May 30;35(10):4365–77. doi: 10.1111/jocn.70383 (PMC13569344; doi:10.1111/jocn.70383)
Supplement: Supplementary file 2 [file JOCN-35-4365-s002.docx]

*Original Article*

**Supplementary Materials**

Additional statistical analyses supporting the findings of the study “Health-related quality of life of adults with Long COVID: a cross-sectional study in primary care”.

**Supplementary Appendix A**. Absolute frequencies and proportions (%) of respondents reporting problems in each EQ-5D-5L dimension.

| **Dimensions** | **Levels** | **All** | | **With**  **Long COVID** | | **Without**  **Long COVID** | | ***pᵃ*** | ***Effect sizeᵇ*** |
| --- | --- | --- | --- | --- | --- | --- | --- | --- | --- |
|  |  | n=226 | | n=156 | | n=70 | |  |  |
|  |  | n | % | n | % | n | % |  |  |
| Mobility | none | 146 | 64.6 | 88 | 56.4 | 58 | 82.9 | <0.001 | 0.31 |
|  | slight | 48 | 21.2 | 38 | 24.4 | 10 | 14.3 |  |  |
|  | moderate | 25 | 11.1 | 24 | 15.4 | 1 | 1.4 |  |  |
|  | severe | 7 | 3.1 | 6 | 3.8 | 1 | 1.4 |  |  |
|  | extreme | 0 | 0 | 0 | 0 | 0 | 0 |  |  |
| Self-care | none | 207 | 91.6 | 140 | 89.7 | 67 | 95.7 | 0.238 | 0.09 |
|  | slight | 10 | 4.4 | 7 | 4.5 | 3 | 4.3 |  |  |
|  | moderate | 8 | 3.5 | 8 | 5.1 | 0 | 0 |  |  |
|  | severe | 1 | 0.4 | 1 | 0.6 | 0 | 0 |  |  |
|  | extreme | 0 | 0 | 0 | 0 | 0 | 0 |  |  |
| Usual activities | none | 159 | 70.4 | 97 | 62.2 | 62 | 88.6 | <0.001 | 0.29 |
|  | slight | 34 | 15.0 | 27 | 17.3 | 7 | 10.0 |  |  |
|  | moderate | 23 | 10.2 | 23 | 14.7 | 0 | 0 |  |  |
|  | severe | 10 | 4.4 | 9 | 5.8 | 1 | 1.4 |  |  |
|  | extreme | 0 | 0 | 0 | 0 | 0 | 0 |  |  |
| Pain/Discomfort | none | 108 | 47.8 | 60 | 38.5 | 48 | 68.6 | <0.001 | 0.27 |
|  | slight | 58 | 25.7 | 43 | 27.6 | 15 | 21.4 |  |  |
|  | moderate | 57 | 25.2 | 50 | 32.1 | 7 | 10.0 |  |  |
|  | severe | 0 | 0 | 0 | 0 | 0 | 0 |  |  |
|  | extreme | 3 | 1.3 | 3 | 1.9 | 0 | 0 |  |  |
| Anxiety/Depression | none | 100 | 44.2 | 52 | 33.3 | 48 | 68.6 | <0.001 | 0.30 |
|  | slight | 56 | 24.8 | 42 | 26.9 | 14 | 20.0 |  |  |
|  | moderate | 47 | 20.8 | 39 | 25.0 | 8 | 11.4 |  |  |
|  | severe | 19 | 8.4 | 19 | 12.2 | 0 | 0 |  |  |
|  | extreme | 4 | 1.8 | 4 | 2.6 | 0 | 0 |  |  |
| ᵃ p-values derived from chi-square tests; statistical significance set at p < 0.05  ᵇ Effect sizes reported as Cramér’s V. | | | | | | | | | |

**Supplementary Appendix B.** Receiver operating characteristic (ROC) analysis of the performance of the US value set for binary EQ-5D-5L dimensions.

US value set	Reference line

**Supplementary Appendix C.** Receiver operating characteristic (ROC) analysis outcomes for the US value set.

| **EQ-5D** | **AUC** | **SE** | **95% CI** | **p-value** | **Gini-index** | **Max K-S metric** |
| --- | --- | --- | --- | --- | --- | --- |
| Mobility | 0.915 | 0.018 | 0.880-0.950 | <0.001 | 0.830 | 0.761 |
| Self-care | 0.896 | 0.028 | 0.841-0.950 | <0.001 | 0.792 | 0.705 |
| Usual activities | 0.905 | 0.019 | 0.868-0.943 | <0.001 | 0.811 | 0.666 |
| Pain/Discomfort | 0.898 | 0.021 | 0.857-0.938 | <0.001 | 0.795 | 0.704 |
| Anxiety/Depression | 0.843 | 0.027 | 0.790-0.895 | <0.001 | 0.685 | 0.599 |
| Abbreviations: AUC -the area under the curve, SE-standard error, CI-confidence interval, Max K-S metric—the maximum Kolmogorov–Smirnov statistic. | | | | | | |

**Supplementary Appendix D.** Crude and adjusted odds ratios for EQ-5D-5L dimensions by Long COVID status.

| **Dimension** | | **All** | | **With Long COVID** | | **Without Long COVID** | | **Crude** | | | **Adjustedᵃ** | | |
| --- | --- | --- | --- | --- | --- | --- | --- | --- | --- | --- | --- | --- | --- |
|  |  | N=226 | | N=156 | | N=70 | |  |  |  |  |  |  |
|  |  | **n** | **%** | **n** | **%** | **n** | **%** | **OR** | **95% CI** | **p-value** | **aOR** | **95% CI** | **p-value** |
| Mobility | none | 146 | 64.6 | 88 | 56.4 | 58 | 82.9 | 1.42 | 0.585-3.421 | 0.441 | 1.17 | 0.460-2.998 | 0.738 |
|  | any | 80 | 35.4 | 68 | 43.6 | 12 | 17.1 |  |  |  |  |  |  |
| Self-care | none | 207 | 91.6 | 140 | 89.7 | 67 | 95.7 | 0.76 | 0.187-3.105 | 0.705 | 0.59 | 0.134-2.595 | 0.485 |
|  | any | 19 | 8.4 | 16 | 10.3 | 3 | 4.3 |  |  |  |  |  |  |
| Usual activities | none | 159 | 70.4 | 97 | 62.2 | 62 | 88.6 | 2.25 | 0.873-5.778 | 0.093 | 2.68 | 1.010-7.134 | 0.048 |
|  | any | 67 | 29.6 | 59 | 37.8 | 8 | 11.4 |  |  |  |  |  |  |
| Pain/Discomfort | none | 108 | 47.8 | 60 | 38.5 | 48 | 68.6 | 2.16 | 1.061-4.385 | 0.034 | 2.24 | 1.064-4.734 | 0.034 |
|  | any | 118 | 52.2 | 96 | 61.5 | 22 | 31.4 |  |  |  |  |  |  |
| Anxiety/depression | none | 100 | 44.2 | 52 | 33.3 | 48 | 68.6 | 3.13 | 1.641-5.980 | <0.001 | 3.86 | 1.927-7.745 | <0.001 |
|  | any | 125 | 55.3 | 104 | 66.7 | 22 | 31.4 |  |  |  |  |  |  |
| Abbreviations: OR-odds ratio; aOR-adjusted odds ratio;  ᵃ- Adjusted for sex, age, and comorbidities. | | | | | | | | | | | | | |
